# The Association between Cardiovascular Disease Risk Factors and 25-Hydroxivitamin D and Related Analytes among Hispanic/Latino Adults: A Pilot Study

**DOI:** 10.3390/nu11081959

**Published:** 2019-08-20

**Authors:** Ramon A. Durazo-Arvizu, Reyna L. Pacheco-Dominguez, Christopher T. Sempos, Holly Kramer, Andrew N. Hoofnagle, Amber Pirzada, Richard S. Cooper, Martha L. Daviglus

**Affiliations:** 1 Department of Public Health Sciences, Loyola University Chicago, Maywood, IL 60153, USA; 2 Centro de Investigación en Políticas, Población y Salud, Universidad Nacional Autónoma de México, México City 04510, Mexico; 3 Vitamin D Standardization Program (VDSP), 520 Ferdinand Dr, Havre de Grace, MD 21078, USA; 4 Department of Laboratory Medicine, Washington University School of Medicine, Seattle, WA 98185, USA; 5 Institute for Minority Health Research, University of Illinois at Chicago, Chicago, IL 60612, USA

**Keywords:** vitamin D, cardiovascular disease risk factors, free vitamin D, Hispanics/Latinos

## Abstract

Although the association of vitamin D levels with cardiovascular risk profiles among Hispanics/Latinos has been studied, little is known about this association among Hispanics/Latinos with chronic conditions. This pilot study determined serum vitamin D and parathyroid hormone (PTH) levels in a sample of participants from the University of Illinois at the Chicago Cohort of Patients, Family and Friends (UIC Cohort) and examined their association with traditional cardiovascular disease risk factors. From July 2012 to June 2016, the UIC Cohort study enrolled and conducted clinical examinations on men and women ages 18 years and older, who had one or more diagnosed chronic diseases/conditions (excluding cancer). This pilot study sample included 40 participants from the six main Hispanic/Latino background groups in the United States, namely Dominican, Cuban, Puerto Rican, Mexican, Central American, and South American, and were grouped by Caribbean or mainland origin. No substantial differences were noted in the vitamin D-related measures by Hispanic/Latino background, but the PTH levels were somewhat higher in the Caribbean vs. mainland group (43.0 ± 4.6 vs. 38.6 ± 2.7 pg/mL). The associations between selected CVD risk factors (systolic and diastolic blood pressure (SBP, DBP), total cholesterol, glucose) and PTH and vitamin D-related analytes were investigated using interval-censored multivariate regression models adjusted for age, sex, percent body fat, serum albumin/calcium, and Hispanic/Latino background. A negative association between total 25[OH]D and blood pressure was corroborated (SBP: β = −1.2, 95%CI = −2.0, −0.3; DBP: β = −0.7, 95% CI = −1.2, −0.1), whereas a positive association with total cholesterol was observed (β = 1.9, 95% CI = 0.02, 3.7). Levels of 1, 25[OH]_2_D were not associated with CVD risk factors, whereas 24, 25[OH]_2_D_3_ was associated with blood pressure (SBP: β = −13.0, 95% CI = −20.7, −5.2; DBP: β = −6.3, 95% CI = −11.6, −1.0). Estimated free 25[OH]D was inversely associated with both SBP (β = −3.5, 95% CI = −6.1, −0.9) and DBP (β = −2.1, 95% CI = −3.8, −0.3). Similarly, calculated bioavailable 25[OH]D was inversely associated with both SBP (β = −9.2, 95% CI = −15.9, −2.4) and DBP(β = −5.3, 95% CI = −9.8, −0.8). In conclusion, a negative association between 25[OH]D with BP was observed and a positive association with lipids is suggested. Due to the small sample size, most associations did not reach statistical significance.

## 1. Introduction

The vitamin D-endocrine system has been shown to play a key role in bone mineral metabolism via the regulation of calcium homeostasis. In addition, an ample range of regulatory functions have been suggested, based on the expression of vitamin D receptors in most tissues and cells of the human body. Associations of 25-hydroxyvitamin D (25[OH]D, the main biomarker of vitamin D status) with chronic diseases such as diabetes [1,2], cardiovascular disease (CVD) [3,4], cancer [5,6,7,8,9], autoimmune diseases [10,11,12,13,14], and hypertension [15,16,17,18,19] have all been reported. These conditions tend to disproportionately affect minority ethnic groups, in particular African-Americans and Hispanics/Latinos. Furthermore, 25[OH]D levels are markedly lower among African-Americans and Hispanics/Latinos when compared to non-Hispanic Whites in the US [20].

Cardiovascular disease and cancer remain the leading causes of death in the United States [21], accounting for over 45% of total deaths. The main biomarker of vitamin D status, 25[OH]D, and related analytes such as 1, 25[OH]D and vitamin D binding globulin, have been associated with CVD risk factors and CVD events [3,22,23]. In the United States, prevalence of vitamin D deficiency (total 25[OH]D < 12 ng/mL) or inadequacy (12–16 ng/mL) [24] is highest among non-Hispanic Blacks (32% and 41%, respectively) and Mexicans (9% and 33%, respectively) [20]. The contribution of vitamin D inadequacy and deficiency to the development and progression of chronic diseases remains an important research question. In particular, the impact of vitamin D levels on the cardiovascular risk factors among Hispanics/Latinos has not been investigated, despite their high burden of adverse CVD risk factors and low levels of 25[OH]D.

Findings from the Hispanic Community Health Study/Study of Latinos have shown that the distribution of the major CVD risk factors differs by Hispanic/Latino background, with Puerto Ricans experiencing the highest prevalence of adverse cardiovascular profiles [25]. The African admixture, which has been associated with lower levels of total 25[OH]D, is largest among Puerto Ricans and Dominicans [26]. The contribution of vitamin D inadequacy and deficiency to the development and progression of chronic diseases and to the racial/ethnic disparity remains an important research challenge. In particular, the association of vitamin D levels with cardiovascular risk factors has not been investigated among diverse Hispanics/Latinos.

Differences in levels of 25[OH]D across ethnic groups and Hispanic/Latino background may be attributed to differences in vitamin D binding globulin (VDBG), the main mode of transport of 25[OH]D and bioavailable 25[OH]D (not bound to VDGB, ~9% bound to albumin, and <1% in the free form). It has been suggested that free 25[OH]D and bioavailable 25[OH]D may be more strongly related to bone density and serum calcium levels than total 25[OH]D [27] and thus may be more sensitive markers of vitamin D status.

A sample of Hispanic/Latino individuals participating in the University of Illinois at the Chicago Cohort of Patients, Family, and Friends (UIC Cohort) was selected for the measurement of 25[OH]D and related analytes including VDBG and free 25[OH]D to study their association with selected traditional CVD risk factors such as blood pressure, lipids, and glucose in this population.

## 2. Methods

### 2.1. Study Population and Data Collection

The UIC Cohort aimed to examine the effects of numerous socioeconomic, sociocultural, biologic, and lifestyle factors on chronic disease management and outcomes in racial/ethnic minority groups. From July 2012 to June 2016, the study enrolled and conducted clinical examinations on 3826 men and women, aged 18 years and older, who had one or more diagnosed chronic diseases/conditions (excluding cancer). The cohort largely consisted of persons from racial/ethnic minority groups (approximately 70% Hispanic/Latino or African-American) and was drawn from the inpatient/outpatient populations (and their families and friends) of the UIC Medical Center and Miles Square in Chicago, IL. Participants underwent a comprehensive physical examination and completed questionnaires to assess medical history, demographic and socioeconomic factors, medical history, lifestyle factors, anthropometric and biological risk factors, biomarkers, nutritional factors, cognitive function, and physical performance. Biological specimens including blood and urine were also obtained. The collection of biological samples and questionnaires followed standardized protocols developed for the Hispanic Community Health Study/Study of Latinos; HCHS/SOL), which are described elsewhere [28]. Briefly, at the examination, the participants’ weight was measured to the nearest 0.1 kg and their height to the nearest centimeter. Body mass index (BMI) was calculated as weight in kilograms divided by height in meters squared. The participants’ body composition (weight, fat mass, lean body mass, and percent body fat) was measured by bioelectrical impedance method with the Tanita Body Composition Analyzer. An automatic sphygmomanometer was used to obtain three seated blood pressure (BP) measurements following a 5-min resting period. Fasting blood samples were collected and measurements of plasma glucose (using a hexokinase enzymatic method, Roche Diagnostics), total serum cholesterol (oxidase enzymatic method), high-density lipoprotein (HDL) cholesterol (direct magnesium dextran sulfate method), and hemoglobin A1c (Tosoh G7 Automated HPLC Analyzer, Tosoh Bioscience) were obtained. Low-density lipoprotein (LDL) cholesterol was estimated via the Friedewald equation [29]. Serum albumin and calcium were determined by an automated clinical chemistry analyzer (Beckman AU680 or AU5815). All subjects gave their informed consent for inclusion before they participated in the study. The study was conducted in accordance with the Declaration of Helsinki, and the protocol was approved by the Institutional Review Board (IRB) of the University of Illinois Chicago (UIC) on March 26, 2018 (Protocol # 2012-0482; “UIC Cohort of Families and Friends Study”).

### 2.2. Pilot Study

To demonstrate the need for a larger study, a pilot study was conducted on a randomly-selected sample of 40 participants from the UIC cohort to determine the serum levels of 25-hydroxyvitamin D (25[OH]D), related metabolites (1, 25[OH]D, 24, 25[OH]D, free 25[OH]D), and parathyroid hormone (PTH). The sample was chosen to include a similar number of individuals from each of the six main Hispanic/Latino groups in the United States, namely Dominican, Central American, Cuban, Mexican, Puerto Rican, and South American. Only participants with complete information on CVD risk factors, and enough serum available for vitamin D measurement were included. A sample size estimate for the study was obtained using the methodology proposed by David Schoenfeld [30]. We used a one-sided Fisher’s Z-test for correlations with 80% statistical power and a 15% type I error rate to detect linear correlations between 25[OH]D and blood pressure of 0.3 or larger.

### 2.3. Quantification of 25[OH]D, 1, 25[OH]D, and 24, 25[OH]D.

Measurements of these analytes were carried out in serum using an immune-affinity enrichment method. Serum or plasma, calibrators, and controls (400 µL) were spiked with deuterated internal standards and purified using anti-1α,25-dihydroxyvitamin D beads from ALPCO. After incubation, the beads were washed and bound analytes eluted with organic solvent. The eluent was dried down and the residue reconstituted with the derivatizing agent 4-phenyl-1,2,4-triazole-3,5-dione (PTAD) in acetonitrile. After incubation at room temperature, the reaction was quenched with water. A portion of the mixture was analyzed on a Waters Xevo TQ tandem mass spectrometer equipped with an Acquity UPLC. Analytes included 25[OH]D_2_, 25[OH]D_2_, 24, 24[OH]_2_D_3,_ 1,25[OH]D_2_, and 1,25[OH]D_3_ with deuterated internal standards for each analyte included. Standards were prepared with stripped human serum. The ability to multiplex the analyses was facilitated by the non-specific binding of multiple vitamin D metabolites from the ALPCO beads. Total 25[OH]D and 1,25[OH]D was calculated as the sum of 25[OH]D_2_, 25[OH]D_3_, and 1,25[OH]D_2_, 1,25[OH]D_3_, respectively. The imprecision of the assay (N = 16 batches, over 10 months, two QC materials per batch) was 9.0–9.4% for 1, 25(OH)_2_D_2_ at 13.6 to 34.4 pg/mL, 14.3–15.0% for 1,25(OH)_2_D_3_ at 18.0 to 43.9 pg/mL, 4.0–6.9% for 24, 25(OH)_2_D_3_ at 1.3 to 4.3 ng/mL, 6.7–8.2% for 25(OH)D_2_ at 9.1 to 27.1 ng/mL, and 3.9–7.2% for 25(OH)D_3_ at 9.4 to 31.2 ng/mL.

### 2.4. Quantification of Vitamin D Binding Globulin

Serum or plasma, calibrators, and controls (10 µL) were denatured, reduced, and alkylated before being proteolytically digested with trypsin. Peptides that were liberated from vitamin D binding globulin (in addition to spiked internal standard peptides) were specifically quantified using liquid chromatography-tandem mass spectrometry and compared with a calibration curve to determine the concentration of protein in the sample. Other peptides were monitored in order to assign the polymorphism present in the sample, which correctly assigned the genotype 97% of the time [31,32].

### 2.5. Estimation of Free 25[OH]D and Bioavailable 25[OH]D

Affinity binding constants between 25[OH]D and VDBG ($K_{vdbg}=7.0\times 10^{8}$) and albumin ($K_{albumin}=6.0\times 10^{5}$) were applied to estimate concentrations of free 25[OH]D as follows:

$$\mathrm{Free}25\left[\mathrm{OH}\right]D=\frac{-b+\sqrt{b^{2}-4ac}}{2a}$$

where

$a=K_{albumin}\times K_{vdbg}\times albumin+K_{vdbg}$

$b=\left(K_{vdbg}*VDBG\right)-\left(K_{vdbg}\times 25\left[\mathrm{OH}\right]D\right)+K_{albumin}\times albumin+1$

c=−25[OH]D

Bioavailable 25[OH]D was then estimated by $(K_{albumin}\times albumin+1)\times \left(\mathrm{Free}\;25\left[\mathrm{OH}\right]D\right)$ [33]. The mathematical formulation using VDBG concentration and affinity for the estimation of free 25[OH]D has been described by Chun et al. [34]. An in-depth explanation of the calculation of free 25[OH]D and bioavailable 25[OH]D is provided by Powe et al. in a supplementary appendix [35].

### 2.6. Measurement of Free 25-Hydroxyvitamin D

An enzyme-linked immunosorbent assay (ELISA) two-step immunoassay procedure was used to measure free 25[OH]D in serum. The ELISA kit was developed by Future Diagnostics using monoclonal antibodies patented by DIAsource [36,37] and is marketed by DIASource. The procedure has a 1.9 pg/mL sensitivity and yields values in the 0–40 pg/mL range. The samples were processed by the University of Washington Department of Laboratory Medicine. Serum samples were measured in duplicate, resulting in a 3.5% coefficient of variation.

### 2.7. Quantification of Intact Parathyroid Hormone

Intact PTH (iPTH) was measured in serum samples using the Beckman-Coulter Dx1 or Access2 automated sandwich immunoassay analyzer [38], which requires 75 µL of the sample. One antibody in the sandwich was directed toward the amino terminus and the other near the end of the carboxy terminus; detection was via chemiluminescence.

### 2.8. Calibration of 25[OH]D Measurements

Concentrations of 25[OH]D in serum using a novel liquid-liquid extraction (LLE) method were standardized to a liquid chromatography-tandem mass spectrometry (LC-MS/MS) approach previously standardized to the National Institute of Standards and Technology (NIST) reference measurement procedure. Briefly, an external sample of 82 men and women from Ghana and the United States with 25[OH]D determination using both the LC-LC/MS and LLE were used to estimate a calibration equation by applying methodology developed by the Vitamin D Standardization Program (VDSP) [39,40]. This resulted in the following standardization equation:

LC−MS/MS=0.868+0.897×LLE

### 2.9. Statistical Analysis

Means and standard deviations were calculated for continuous measurements, and proportions for categorical variables. Scatter plots and other graphical analyses were performed to ascertain the need for variable transformation to achieve better-fit regression models. The distributions of all continuous variables of interest were estimated via kernel density estimators with the Epanechnikov kernel function [41]. Analysis of variance for continuous variables and χ^2^ test for categorical measures were used to compare values across Hispanic/Latino groups. Pearson’s correlation coefficient was applied to assess the associations between each CVD risk factor (e.g., systolic BP) and 25[H]D or a related metabolite. We then fit multivariable censored linear regression analysis [42], adjusting for age, sex, percent body fat, serum albumin, serum calcium, PTH, and Hispanic/Latino background. This approach is preferred over multivariate linear regression when the outcome variable (i.e., blood pressure, total cholesterol) cannot be observed for participants under certain conditions (in this case, medication use). Participants undergoing treatment for hypertension (15%), diabetes (10%), or hypercholesterolemia (15%) had their measured values of systolic BP (SBP), diastolic BP (DBP), fasting glucose, HbA1c, total-, LDL- and HDL-cholesterol, and triglycerides censored at the observed value. There are several approaches for analyzing quantitative traits such as blood pressure, cholesterol, and glucose when the participant is on medication. Conventional approaches that include (a) ignoring medication use and performing a traditional regression analysis, (b) including a binary indicator for treatment in regression model, and (c) excluding treated participants altogether. Simulation studies have demonstrated that censored normal regression approaches not only enjoy greater statistical power, but yield less bias estimators [43]. STATA 15.1 (College Station, TX, USA) was used for all analyses.

## 3. Results

**Figure 1:**
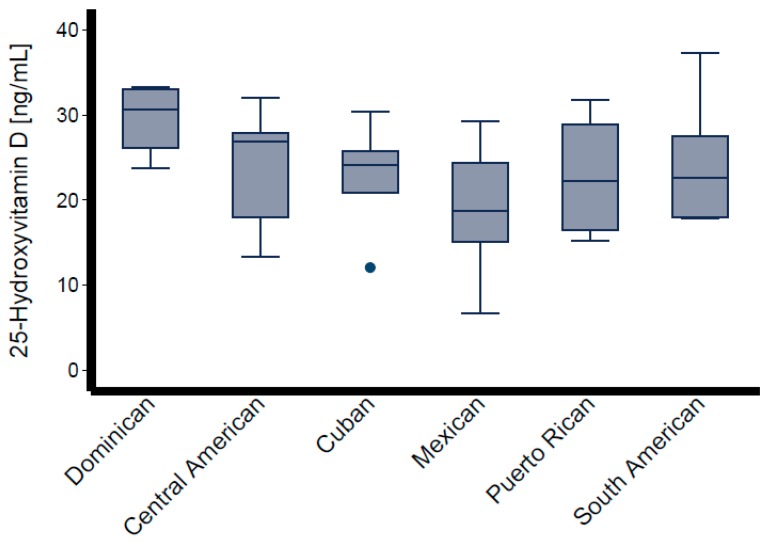
Box-plot distributions of 25-Hydroxyvitamin D (25[OH]D) by Hispanic/Latino group. The dot indicates an outlier (25[0H]D = 11.5 ng/mL).

**Figure 2:**
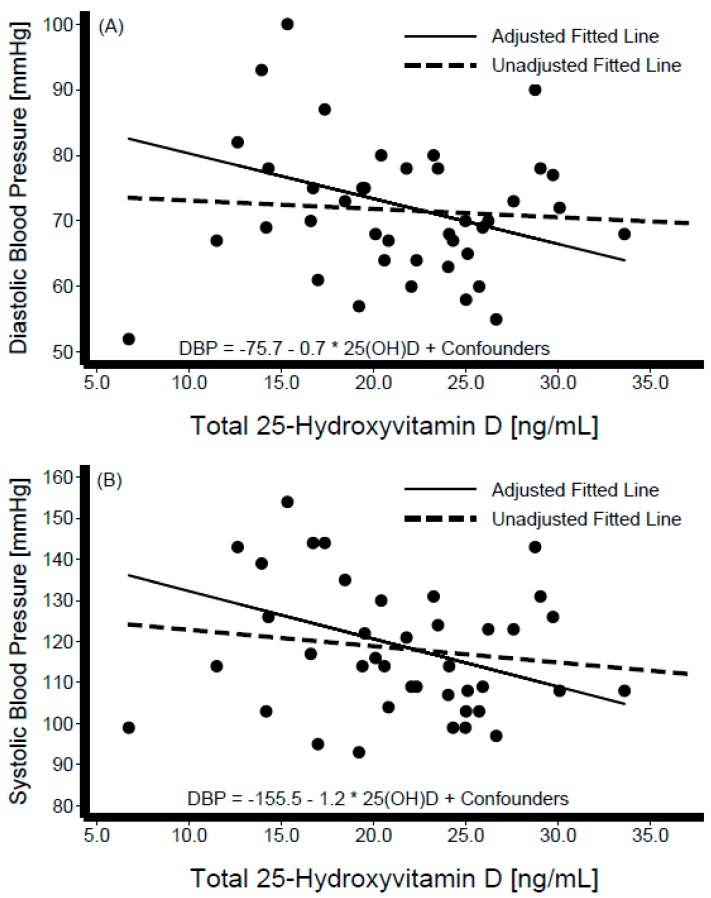
Scatter diagram and best-fit regression line of total 25-Hydroyvitamin D (25[OH]D) with diastolic blood pressure (panel **A**) and systolic blood pressure (panel **B**). Interval-censored regression models were adjusted for age, sex, percent body fat, serum albumin, serum calcium, parathyroid hormone, and Hispanic/Latino background.

**Figure 3:**
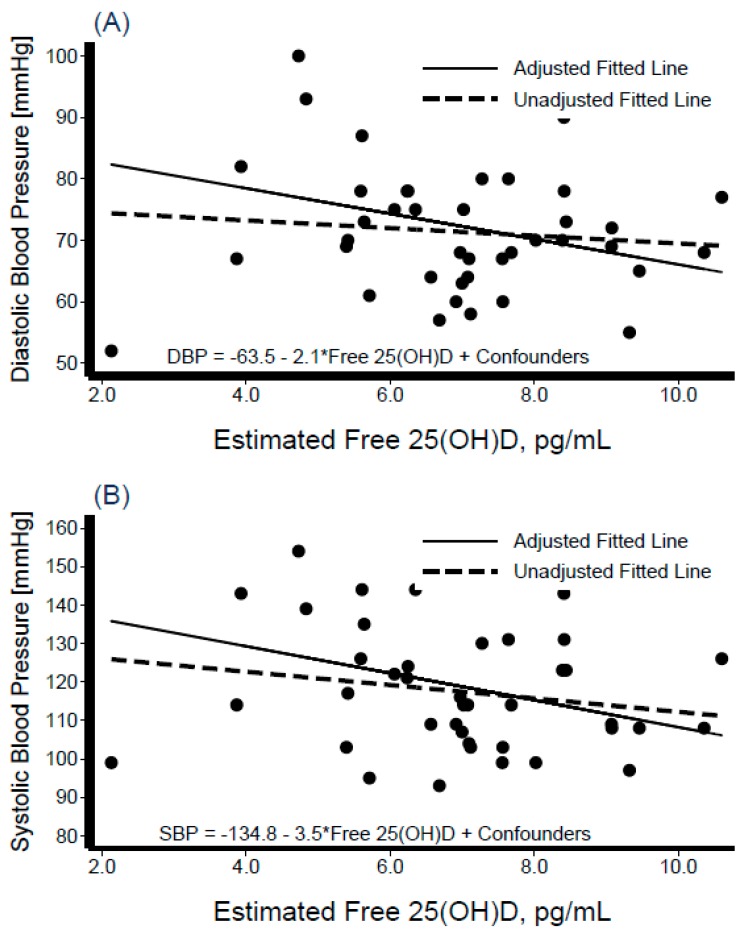
Scatter diagram and best-fit regression line of estimated free 25-Hydroxyvitamin D (25[OH]D) with diastolic blood pressure (panel **A**) and systolic blood pressure (panel **B**). Interval-censored regression models were adjusted for age, sex, percent body fat, serum albumin, serum calcium, parathyroid hormone, and Hispanic/Latino background.

**Figure 4:**
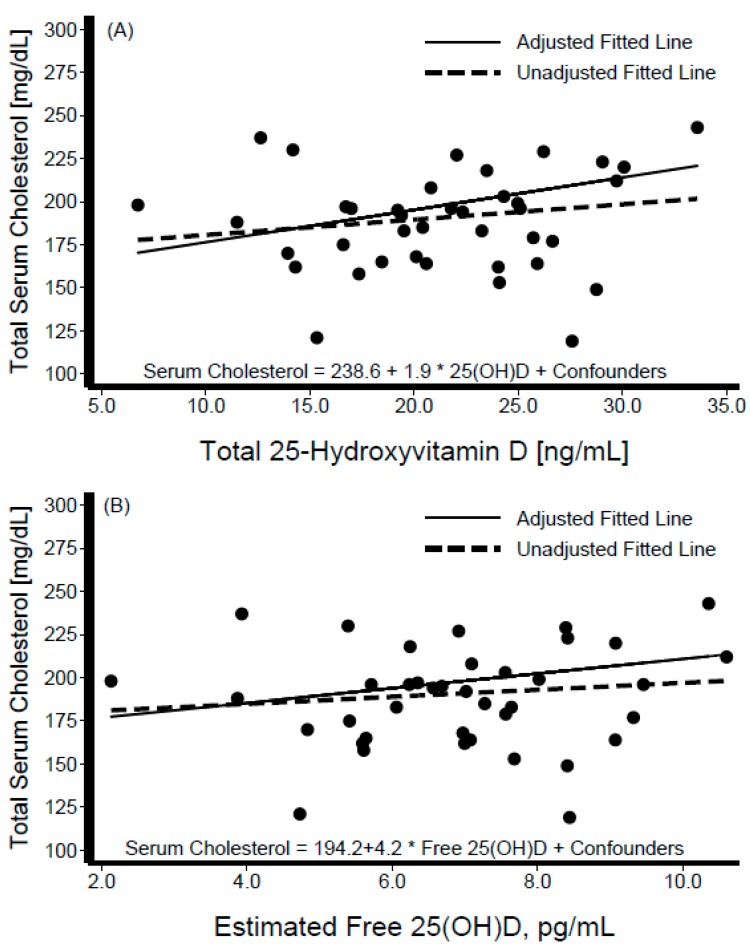
Scatter diagram and best-fit regression line of total serum cholesterol against total 25-hydroxyvitamin D (panel **A**) and estimated free 25-Hydroxyvitamin D (panel **B**). Interval-censored regression models were adjusted for age, sex, percent body fat, serum albumin, serum calcium, parathyroid hormone, and Hispanic/Latino background.

Descriptive characteristics of the participants are shown in Table 1, stratified by Hispanic/Latino background. The sample was composed of 40 participants from Dominican (*n* = 4), Central American (*n* = 8), Cuban (*n* = 7), Mexican (*n* = 7), Puerto Rican (*n* = 7), and South American (*n* = 7) backgrounds; of these 19 (48%) were women. The variables of interest (Table 1) did not differ significantly across Hispanic/Latino groups. The average BMI was 31 kg/m^2^ overall and varied from 28 kg/m^2^ among persons of Central American background to 35 kg/m^2^ among those of Cuban background. Mean blood pressure levels were SBP = 117.5 ± 15.71 mmHg and DBP = 71.4 ± 10.27 mmHg overall, and were the lowest among individuals from a Mexican background (SBP = 109 mmHg, DBP = 65 mmHg), and highest among those from a Puerto Rican background (SBP = 130 mmHg, DBP = 80 mmHg). Mean total cholesterol was 190.8 ± 33.42 mg/dL overall, highest among those with Central American backgrounds (206.0 ± 44.3 mg/dL), and lowest among those with a Puerto Rican background (173.4 ± 35.7 mg/dL). Mean total serum 25[OH]D level was 21.5 ± 5.8 ng/mL overall, and highest among persons of Dominican background (26.9 ± 3.9 ng/mL) and lowest among those of Mexican background (17.8 ± 6.4 ng/mL). The average PTH in this sample was 49.3 ± 18.2 pg/mL, with Mexican individuals having the lowest mean levels (32.1 ± 8.5 pg/mL) and those with a Dominican background showed the highest (44.1 ± 7.9 pg/mL) (Table 1). Figure 1 depicts the box-plot distributions of 25[OH]D by Hispanic/Latino group. Figure 2 illustrates the associations of total 25[OH]D with DBP (panel A) and SBP (panel B). Despite substantial variability in BP levels, we observed a negative association between these levels and 25[OH]D. Similarly, the graphical representation of the association between levels of estimated free 25[OH]D and BP is shown in Figure 3. The unadjusted relationship, as illustrated by the linear regression curves, between free 25[OH]D and blood pressure appeared to be weaker. The top panel (panel A) of Figure 4 shows the association between total 25[OH]D and total serum cholesterol, whereas panel B describes the estimated free 25[OH]D-total serum cholesterol association. A modest positive linear association can be seen from the adjusted and unadjusted regression lines in the figure.

**Figure 5:**
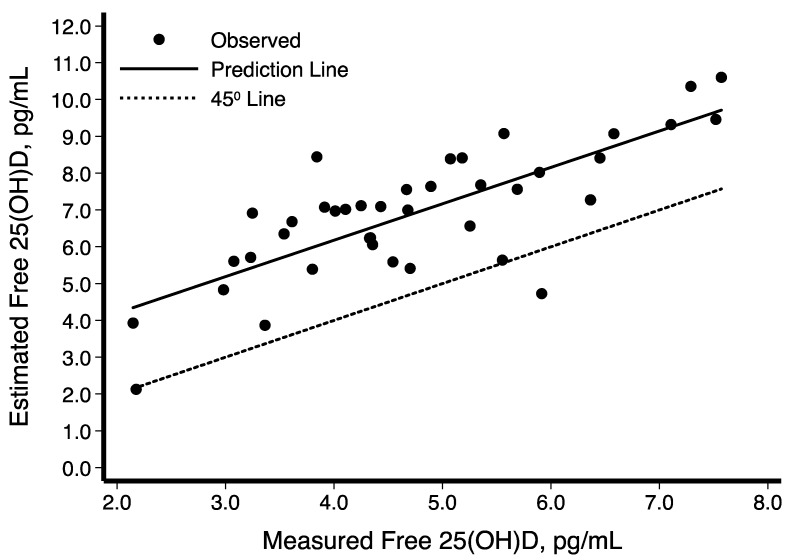
Scatter diagram and best-fit regression line of the estimated free 25-Hydroxyvitamin D (25[OH]D) with measured free 25[OH]D (DiaSource Immuno-Assays, S.A.; Louvain-La-Neuve, Belgium).

**Figure 6:**
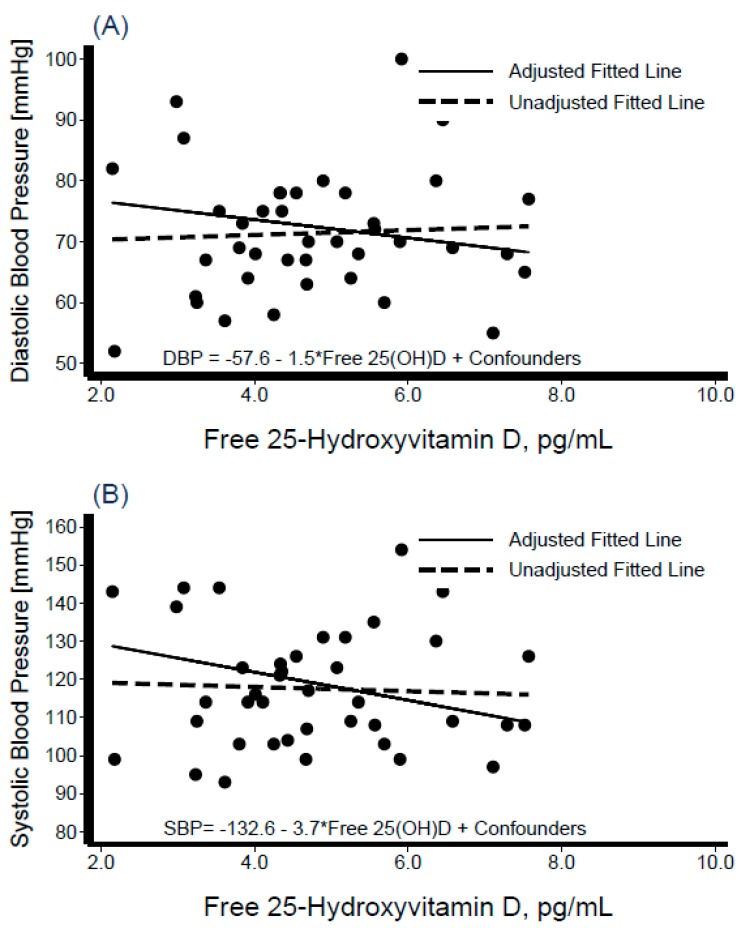
Scatter diagram and best-fit regression line of measured free 25-Hydroxyvitamin D (DiaSource Immuno-Assays, S.A.; Louvain-La-Neuve, Belgium) with diastolic blood pressure (panel **A**) and systolic blood pressure (panel **B**). Interval-censored regression models were adjusted for age, sex, percent body fat, serum albumin, serum calcium, parathyroid hormone, and Hispanic/Latino background.

Parameter estimates (β coefficient representing the change in the outcome per unit change in the 25[OH]D or related analyte) and corresponding 95% confidence intervals of multivariate regression models are presented in Table 2. A negative association between total 25[OH]D and BP was observed (SBP: β = −1.2, 95%CI = −2.0, −0.3; DBP: β = −0.7, 95% CI = −1.2, −0.1), in addition to a positive association with total cholesterol (β = 1.9, 95% CI = 0.02, 3.7). Levels of 1, 25[OH]D were not associated with the CVD risk factors of interest, whereas 24, 25[OH]D was associated with BP (SBP: β = −13.0, 95% CI = −20.7, −5.2; DBP: β = −6.3, 95% CI = −11.6, −1.0) and triglycerides (β = 31.3, 95% CI = 3.5, 59.1). Higher VDBG was associated with higher levels of HDL-cholesterol (HDL) (β = 0.2, 95% CI = 0.1, 0.4). Estimated free 25[OH]D was associated with both SBP (β = −3.5, 95% CI = −6.1, −0.9) and DBP (β = −2.1, 95% CI = −3.8, −0.3), as was bioavailable 25[OH]D(SBP: β = −9.2, 95% CI = −15.9, −2.4; DBP: β = −5.3, 95% CI = −9.8, −0.8). A negative correlation was noted between PTH and 25[OH]D (−0.14), 24, 25[OH]_2_D_3_ (−0.13), estimated free 25[OH]D (−0.12) and bioavailable 25[OH]D (−0.17) (data not shown). Figure 5 demonstrates the concordance of estimated and directly measured levels of free 25[OH]D. The mean concentrations and variability of directly measured free and bioavailable 25[OH]D were smaller than the corresponding estimated values (Table 1). The associations between free (bioavailable) 25[OH]D and CVD risk factors were similar for both the estimated and directly measured levels. Figure 6 illustrates the association between diastolic (panel A) and systolic (panel B) blood pressure and measured levels of free 25[OH]D. Despite the observed similarity, statistical significance between measured free (bioavailable) 25[OH]D and DBP was not maintained.

Overall, a negative statistically significant association with BP and a consistent positive, albeit not statistically significant association with lipids (Total/HDL/non-HDL/triglycerides/LDL), 25[OH]D, and related analytes was observed (except for 1, 25[OH]D).

## 4. Discussion

We observed an inverse association between levels of 25[OH]D and related analytes with systolic and diastolic blood pressure, and a positive association with lipids (triglycerides, HDL/non-HDL/LDL/total cholesterol), consistent with previous reports on non-Hispanic individuals [44,45,46,47,48,49,50,51,52,53].

Vimaleswaran et al. [50] meta-analyzed data from over 100,000 individuals of European ancestry from 35 cohorts in the D-CarDia collaboration study. They reported inverse associations of 25(OH)D with both systolic BP and hypertension. Furthermore, they demonstrated a negative association between variants of genes that affected 25[OH]D synthesis or substrate availability and blood pressure, which led to their conclusion that increased 25[OH]D concentrations played a causal role in reducing the risk of hypertension. Data from a US representative sample collected as part of the National Nutrition and Examination Survey (NHANES) 2001 to 2010 were analyzed by Vishnu et al. [51] to ascertain the association between standardized levels of 25[OH]D and BP by sex and race/ethnic group. A statistically significant association was found for non-Hispanic White and non-Hispanic Black females, but only a marginal association for Hispanic males. Scragg [48] reported a negative association between quantiles of 25[OH]D and mean levels of blood pressure in an ethnically diverse population of the United States, which persisted after accounting for body composition. Schmitz et al. [47] investigated whether the inverse relationship between 25[OH]D and BP persisted among 1334 Hispanic and African-Americans using data from the Insulin Resistance Atherosclerosis Family Study and found a significant negative association in both groups. However, additional adjustment for body composition annulled the observed association. Fiscella et al. [46] explored BP and hypertension disparities between non-Hispanic Blacks and non-Hispanic Whites using NHANES 2001–2006 data and found that they could be partially explained by socio-demographic factors, health status, health insurance, health care access, and other biomarkers including hemoglobin A1c(%), C reactive protein, and albumin. An additional 29% reduction in the adjusted difference between these groups was observed after adjusting for 25[OH]D, suggesting that vitamin D may underlie race/ethnic disparities in BP levels independent of better-known socioeconomic drivers.

The published literature offers a mechanistic explanation for the observed association between blood pressure and vitamin D. The renin-angiotensin system has been implicated in the regulation of BP levels as well as the risk of heart attack and stroke. Li [54] postulated that renin expression may be regulated by vitamin D and that deficiency of vitamin D could lead to an elevation in renin expression. His hypothesis is in line with epidemiological and clinical research that imply a negative association between BP and plasma renin activity and vitamin D. Ullab et al. [49] reviewed the evidence relating vitamin D and hypertension and offered support to the hypothesis linking vitamin D with the regulation of the renin-angiotensin system and thus its association with blood pressure.

The association of vitamin D with lipid measures has not been adequately examined among diverse Hispanic/Latino adults [45,53]. A positive association between high density cholesterol and apolipoprotein A-I in a sample of 358 Belgian men and women was reported by Auwerx et al. [44]. A systematic review of the literature examining 22 cross-sectional and 10 placebo-control studies found a positive association between 25[OH]D levels and high-density cholesterol [22]. More recently, Vogt and colleagues [52] used NHANES 2001–2006 data to investigate the association between 25[OH]D with total cholesterol, HDL/LDL cholesterol, and the modifying effect of body composition on this association. Lower and higher levels of HDL- and LDL-cholesterol, respectively, were associated with lower levels of vitamin D among obese individuals, but this relationship was not present in the non-obese. A weaker positive association between total cholesterol and levels of 25[OH]D among the non-obese was found. A recent study has reported a positive association between serum 25[OH]D with indicators of arteriosclerosis and arterial stiffness in an older adult population [49]. In addition, several animal studies have linked excessive vitamin D intake with the presence and development of arteriosclerotic lesions [55,56,57]. Our study suggests a positive association between total cholesterol and 25[OH]D among chronic disease patients with high mean body mass index (30.8 ± 6.4 kg/m^2^). Our results, although not statistically significant, highlight the importance of implementing larger scale studies to investigate the role of large amounts of vitamin D supplementation and serum levels of 25[OH]D in the development of coronary atherosclerosis.

Free 25(OH)D can be directly measured or calculated from one of the equations that have been derived [58]. The accuracy of either method is uncertain, as is the relationship among them. This makes the development of free 25(OH)D clinical cut-off values premature [59,60]. What is needed is a program to harmonize free 25(OH)D measurement in all vitamin D research—both directly measured and calculated—in order to avoid the chaos that currently affects the interpretation of serum total 25(OH)D [61]. The assay used to measure free 25(OH)D is the only kit that is commercially available. Although other assays have been used in vitamin D research, there is currently no widely-accepted reference measurement procedure for the direct measurement of free 25(OH)D. Thus, we focused our presentation of the results and discussion on estimated free 25(OH)D. This approach is based on extensive research on the measurement of vitamin D binding globulin (VDBG) and the mathematical modeling of the estimation of free 25(OH)D based on the concentration of VDBG and albumin and their binding affinity with 25(OH)D. There is an urgent need to harmonize the free 25(OH)D measurement in research, given that clinical cut-offs for interpreting free 25(OH)D values are being proposed. The first step toward this process would be the development of “trueness” controls that could be used to “harmonize” all data collected in vitamin D research, which could LAO be used to harmonize clinical measurements. Furthermore, as illustrated by Figure 5, there is a strong correlation between the directly measured and estimated levels of free 25(OH)D. Thus, our choice to emphasize the association of estimated free 25(OH)D with CVD risk factors will allow vitamin D researchers to compare our findings with a broad range of studies.

Most clinical trials of vitamin D supplementation that have been published to date have essentially demonstrated minimal or no clinical benefit for a wide range of CVD risk factors including hypertension, diabetes, obesity, and incident CVD [62,63,64,65]. However, the majority of the ongoing and completed trials largely included vitamin D-sufficient individuals and some participants with low total 25[OH]D levels [66]. Furthermore, a lack of consensus regarding the definition of vitamin D sufficiency and the role of race/ethnicity in determining vitamin D cut-points have impeded research on the potential benefits of vitamin D supplementation on CVD risk. Our study lends support to the association between vitamin D and blood pressure levels among persons with prevalent chronic disease. Further randomized clinical trials on the role of vitamin D supplementation in improving blood pressure on patients with chronic conditions are needed.

### 4.1. Limitations of the Study

The current study included only a small sample of persons with a pre-existing chronic condition (40 chronic disease patients randomly selected from the UIC Cohort). Although investigators argue the need to perform a formal sample size calculation when the objective of a study is to explore the associations, the selected sample size was justified by applying a statistical approach suggested by Shoenfeld [30] where a larger than “usual” type I error rate is accepted. The aim of the current pilot study was to gather information on the association between vitamin D-related analytes and traditional CVD risk factors in Hispanic/Latino adults with prevalent chronic diseases in order to determine the need for a larger study in this population. Although the sample included participants from the six major Hispanic/Latino groups in the United States, the small sample size could not yield sufficient information to contrast across Hispanic/Latino groups. These groups differ in many important characteristics that could potentially confound the observed associations including African admixture, length of residence in the, U.S.; level of education and other measures of socioeconomic status, access to health services, and occupation. For instance, the small sample size hindered our ability to adjust for pre-existing conditions known to be associated with hypertension and other CVD risk factors such as diabetes mellitus. Parsimonious models were adopted to avoid data over fitting. In this sample, 25[OH)D ranged from 7.0 ng/mL to 34 ng/mL with levels below 12 ng/mL (the most commonly accepted cutoff for vitamin D deficiency) in only 5% of participants. In comparison, 25[OH]D ranged from 5 ng/mL to 99 ng/mL (average 22.6 ng/mL) among Mexican Americans participating in the NHANES 2007–2014 with 25[OH]D levels below 12 ng/mL in 7.0% of Mexican American NHANES participants. It is possible that stronger associations would have been observed in the current study if a higher number of individuals with vitamin D deficiency were included. Nonetheless, our findings on a sample of diverse Hispanic/Latino adults are consistent with previous reports of associations between vitamin D and BP.

Hypervitaminosis D has been linked to adverse health outcomes such as hypercalcemia and nephrolithiasis [24] as well as CVD and CVD risk factors [67,68,69,70]. Despite a dearth of studies examining the safety of vitamin D intake, the Institute of Medicine report concluded that daily intakes in excess of 4000 IU represent the threshold beyond which the harm from vitamin D starts to increase. However, it has been observed that although levels of 10,000 IU could be associated with vitamin D toxicity, daily intakes below this level are unlikely to lead to adverse health outcomes [24]. Thus, caution should be exercised when interpreting the inverse relationship between serum 25(OH)D and related analytes with CVD risk.

### 4.2. Future Directions

Larger studies with sufficient numbers of participants from diverse Hispanic/Latino groups such as the Hispanic Community Health Study/Study of Latinos are needed to further investigate the role of vitamin D in cardiovascular health in the Hispanic/Latino population. Large vitamin D intervention trials including chronic disease patients with low levels of 25[OH]D (e.g., <12 ng/mL) are also needed to enhance our understanding of the role played by vitamin D deficiency on health status. We also need to re-examine the experimental data on the role of vitamin D in atherosclerosis across the entire spectrum of serum 25[OH]D concentrations [69].

### 4.3. Conclusions

Our findings support the potential negative role of vitamin D deficiency on blood pressure in a sample of Hispanic/Latino adults with prevalent chronic diseases. The preliminary findings from this pilot study underscore the need for larger studies with long-term follow-up that include individuals of diverse demographic backgrounds, geographic locations, health statuses, and 25[OH]D levels in order to elucidate the impact of vitamin D on cardiovascular disease risk factors.

## Figures and Tables

**Table 1:** Participant characteristics by Hispanic/Latino background.

	Hispanic/Latino Background [Mean ± SD/*n*(%)]						
	Dominican	Central American	Cuban	Mexican	Puerto Rican	South American	Total
**Sample Size**	4	8	7	7	7	7	40
**Age (years)**	49.8 ± 9.7	41.9 ± 7.5	49.6 ± 2.4	44.9 ± 7.6	47.6 ± 4.1	44.7 ± 5.6	46.0 ± 6.5
**Male (%)**	50%	50%	43%	57%	57%	57%	53%
**BMI (kg/m^2^)**	31.8 ± 3.9	28.2 ± 3.0	35.5 ± 7.7	29.0 ± 5.8	31.6 ± 9.7	29.3 ± 4.6	30.8 ± 6.4
**SBP (mmHg)**	116.0 ± 8.9	118.4 ± 17.7	115.4 ± 12.4	108.7 ± 17.0	130.0 ± 17.3	115.9 ± 13.3	117.5 ± 15.7
**DBP (mmHg)**	74.0 ± 4.2	72.4 ± 12.0	70.0 ± 8.9	64.6 ± 11.4	79.7 ± 11.7	68.7 ± 3.2	71.4 ± 10.3
**Body Fat (%)**	36.7 ± 9.7	30.5 ± 6.8	41.0 ± 9.5	28.9 ± 12.2	28.7 ± 10.0	31.1 ± 8.5	32.5 ± 10.0
**Glucose (mg/dL)**	95.0 ± 12.7	87.5 ± 8.1	91.1 ± 10.2	120.6 ± 59.0	126.7 ± 111.9	83.4 ± 5.7	100.8 ± 53.0
**Total Cholesterol (mg/dL)**	198.0 ± 24.8	206.0 ± 44.3	188.9 ± 34.8	188.1 ± 24.4	173.4 ± 35.7	191.4 ± 29.7	190.8 ± 33.4
**HDL Cholesterol (mg/dL)**	51.3 ± 10.3	44.0 ± 9.3	43.4 ± 6.9	43.6 ± 8.2	50.3 ± 18.1	33.3 ± 6.9	43.8 ± 11.5
**Non-HDL Cholesterol (mg/dL)**	146.8 ± 30.4	162.0 ± 37.1	145.4 ± 30.6	144.6 ± 22.2	123.1 ± 30.4	158.1 ± 29.7	147.1 ± 31.4
**LDL (mg/dL)**	127.0 ± 35.2	130.4 ± 32.0	120.3 ± 28.3	119.6 ± 29.1	104.7 ± 26.2	124.5 ± 25.1	120.9 ± 28.4
**HbA1c (%)**	5.8 ± 0.8	5.4 ± 0.4	5.6 ± 0.3	6.5 ± 2.0	6.2 ± 2.5	5.5 ± 0.3	5.8 ± 1.4
**Triglycerides (mg/dL)**	98.8 ± 43.4	157.9 ± 70.9	125.9 ± 28.7	124.9 ± 81.1	92.4 ± 36.5	168.4 ± 58.4	131.0 ± 60.8
**Total 25(OH)D (ng/mL)**	26.9 ± 3.9	21.9 ± 5.9	20.9 ± 5.0	17.8 ± 6.4	21.1 ± 5.5	22.4 ± 5.9	21.5 ± 5.8
**1, 25(OH)D (pg/mL)**	60.3 ± 13.8	54.0 ± 25.4	57.0 ± 22.2	50.9 ± 7.3	34.5 ± 11.9	43.3 ± 12.0	49.3 ± 18.2
**PTH (pg/mL)**	44.1 ± 7.9	40.4 ± 13.3	41.9 ± 15.8	32.1 ± 8.5	43.5 ± 28.6	42.9 ± 14.6	40.6 ± 16.2
**24, 25(OH)D (pg/mL)**	1.6 ± 0.6	1.2 ± 0.5	0.9 ± 0.3	0.9 ± 0.5	1.2 ± 0.5	1.4 ± 0.9	1.2 ± 0.6
**VDBG ^1^ (µg/mL)**	229.6 ± 25.8	242.7 ± 17.9	229.9 ± 27.2	220.0 ± 20.0	228.1 ± 24.7	213.1 ± 18.1	227.4 ± 22.9
**Estimated Free 25(OH)D ^2^ (pg/mL)**	8.7 ± 1.8	6.6 ± 1.6	6.7 ± 1.4	6.0 ± 2.2	6.8 ± 1.5	7.6 ± 1.7	6.9 ± 1.8
**Bioavailable 25(OH)D ^3^ (ng/mL)**	3.4 ± 0.6	2.5 ± 0.6	2.6 ± 0.6	2.3 ± 0.9	2.5 ± 0.7	3.0 ± 0.8	2.7 ± 0.8
**Measured Free 25(OH)D ^4^ (pg/mL)**	6.0 ± 1.4	4.4 ± 1.3	3.9 ± 0.6	4.1 ± 1.6	5.5 ± 0.8	5.3 ± 1.6	4.8 ± 1.4
**Bioavailable 25(OH)D ^5^ (ng/mL)**	2.3 ± 0.4	1.7 ± 0.5	1.5 ± 0.2	1.5 ± 0.7	2.1 ± 0.5	2.1 ± 0.7	1.8 ± 0.6
**Number with Chronic Condition**							
** High Blood Pressure**	0	0	3	1	4	1	7
** Heart Failure**	0	0	1	0	1	0	2
** Stroke**	0	0	0	1	0	0	1
** Peripheral Arterial Disease**	0	0	0	0	0	1	1
** Heart Burn**	3	3	5	2	4	5	22
** Sleep Disorder**	3	0	1	0	3	0	7
** Hepatitis**	0	0	0	0	1	0	1
** Liver Disease**	0	0	0	0	1	0	1

^1^ Vitamin D binding globulin; ^2^ Estimated Free 25(OH)D; ^3^ From Estimated Free 25(OH)D; ^4^ Measured Free 25(OH)D; ^5^ From Measured Free 25(OH)D.

**Table 2:** Regression coefficient (95% confidence interval) corresponding to a given exposure (25(OH)D analyte) for each outcome (cardiovascular risk factor), adjusted for age, sex, percent body fat, serum albumin, serum calcium, parathyroid hormone, and Hispanic/Latino background. The regression coefficient (β weight) represents the expected change in the average response (e.g., systolic blood pressure) for each unit increase in the vitamin D-related analyte (e.g., 25(OH)D). For instance, a decrease in the average systolic blood pressure of 1.2 mmHg is expected for one ng/mL increase in 25(OH)D.

		CVD Risk Factor						
25(OH)D Analyte	SBP	DBP	Glucose	Total Cholesterol	HDL Cholesterol	Non-HDL ^‡^ Cholesterol	Triglycerides	LDL Cholesterol
**25(OH)D**	−1.2 (−2.0, −0.3) *	−0.7 (−1.2,−0.1) *	−0.1 (−2.6, 2.8)	1.9 (0.02, 3.7) *	0.5 (−0.1, 1.1)	1.4 (−0.3, 3.1)	2.3 (−0.8, 5.4)	1.0 (−0.5, 2.5)
**1, 25(OH)D**	0.1 (−0.2, 0.4)	0.0 (−0.2, 0.2)	−0.4 (−1.2, 0.4)	−0.2 (−0.8, 0.4)	−0.0 (−0.2, 0.2)	−0.2 (−0.8, 0.4)	0.3 (−0.7, 1.2)	−0.3 (−0.7, 0.2)
**24, 25(OH)D**	−13.0 (−20.7,−5.2) *	−6.3 (−11.6,−1.0) *	−12.2 (−35.9, 11.6)	13.1 (−4.9, 31.0)	0.1 (−5.9, 6.2)	13.6 (−2.3, 29.5)	31.3 (3.5, 59.1) *	7.9 (−6.6, 22.3)
**VDBG ^1^**	−0.1 (−0.3, 0.2)	−0.1 (−0.2, 0.1)	0.3 (−0.4, 0.9)	0.4 (−0.1, 0.9)	0.2 (0.1, 0.4) *	0.2 (−0.2, 0.7)	0.1 (−0.8, 0.9)	0.2 (−0.2, 0.6)
**Free 25(OH)D ^2^**	−3.5 (−6.1,−0.9) *	−2.1 (−3.8,−0.3) *	−1.3 (−9.6, 7.0)	4.2 (−1.7, 10.2)	0.7 (−1.2, 2.7)	3.6 (−1.8, 9.0)	6.2 (−3.6, 16.1)	2.5 (−2.3, 7.3)
**Bioavailable 25(OH)D ^3^**	−9.2 (−15.9,−2.4) *	−5.3 (−9.8,−0.8) *	−2.3 (−23.9, 19.2)	11.0 (−4.6, 26.5)	1.7 (−3.4, 6.8)	9.4 (−4.5, 23.3)	16.2 (−9.3, 41.6)	6.5 (−5.9, 18.9)
**Free 25(OH)D ^4^**	−3.7 (−7.3,−0.1) *	−1.5 (−3.9, 0.9)	−2.7 (−13.6, 8.3)	−1.6 (−9.8, 6.6)	−0.3 (−2.9, 2.3)	−1.3 (−8.6, 6.1)	−4.1 (−17.4, 9.3)	−0.1 (−6.6, 6.4)
**Bioavailable 25(OH)D ^5^**	−9.6 (−18.9,−0.3) *	−3.8 (−10.1, 2.5)	−7.6 (−35.9, 20.6)	−4.2 (−25.2, 17.0)	−0.9 (−7.7, 5.8)	−3.0 (−21.9,−15.9)	−11.2 (−45.4, 23.1)	−0.1 (−16.9, 16.6)

* Significant at *p* < 0.05; ^1^ Vitamin D Binding Globulin; ^2^ Estimated Free 25(OH)D; ^3^ Bioavailable from Estimated Free 25(OH)D; ^4^ Measured Free 25(OH)D; ^5^ Bioavailable from Measured Free 25(OH)D. ^‡^ Total Cholesterol–HDL cholesterol.
